# How robust is the language architecture? The case of mood

**DOI:** 10.3389/fpsyg.2013.00505

**Published:** 2013-08-22

**Authors:** Jos J. A. Van Berkum, Dieuwke De Goede, Petra M. Van Alphen, Emma R. Mulder, José H. Kerstholt

**Affiliations:** ^1^Department of Languages, Literature and Communication, Utrecht Institute of Linguistics OTS, Utrecht UniversityUtrecht, Netherlands; ^2^Neurobiology of Language Department, Max Planck Institute for PsycholinguisticsNijmegen, Netherlands; ^3^TNO Human FactorsSoesterberg, Netherlands

**Keywords:** mood, emotion, language comprehension, anticipation, reference, joint attention

## Abstract

In neurocognitive research on language, the processing principles of the system at hand are usually assumed to be relatively invariant. However, research on attention, memory, decision-making, and social judgment has shown that mood can substantially modulate how the brain processes information. For example, in a bad mood, people typically have a narrower focus of attention and rely less on heuristics. In the face of such pervasive mood effects elsewhere in the brain, it seems unlikely that language processing would remain untouched. In an EEG experiment, we manipulated the mood of participants just before they read texts that confirmed or disconfirmed verb-based expectations about who would be talked about next (e.g., that “*David praised Linda because* … ” would continue about Linda, not David), or that respected or violated a syntactic agreement rule (e.g., “*The boys turns*”). ERPs showed that mood had little effect on syntactic parsing, but did substantially affect referential anticipation: whereas readers anticipated information about a specific person when they were in a good mood, a bad mood completely abolished such anticipation. A behavioral follow-up experiment suggested that a bad mood did not interfere with verb-based expectations *per se*, but prevented readers from using that information rapidly enough to predict upcoming reference *on the fly*, as the sentence unfolds. In all, our results reveal that background mood, a rather unobtrusive affective state, selectively changes a crucial aspect of real-time language processing. This observation fits well with other observed interactions between language processing and affect (emotions, preferences, attitudes, mood), and more generally testifies to the importance of studying “cold” cognitive functions in relation to “hot” aspects of the brain.

## Introduction

In cognitive science and neuroscience, we tend to assume—implicitly or explicitly—that the architecture of the mind is relatively invariant. Of course, there are massive changes during development, and as a result of pathology. However, there's a shared idea that somewhere between the promising torrents of childhood and the dangerous waters of old age or disease, the mental faculties of Homo Sapiens are in more quiet waters, involving operational principles that are relatively stable over context and time. In the studies reported below, we examine the stability of one of our species' most distinctive mental faculties: the language architecture.

At some level, the invariance assumption simply has to be correct. If minds were totally variable all of the time, with no stable principles of operation, we'd all have a very hard time predicting and understanding the thoughts, feelings, and actions of our conspecifics. The results would be disastrous: parenting would fail, communication and other types of coordination would be impossible, groups would dismantle, and societies would collapse. At the same time, we know that the mind's mode of operation can to some extent be adjusted even in quiet waters. A familiar example of such functional adjustment is sleep. A perhaps less obvious example in this context, but well-known as a phenomenon to all of us, is mood.

Mood is a relatively slow-changing affective background state, a usually mild positive or negative feeling that is not strongly associated with particular objects or events, and has little cognitive content (Forgas, 1995; Scherer, 2005). Compared to full-blown event-triggered emotions such as rage, exhilaration, or surprise, mood fluctuates less rapidly, and usually less intensely (Scherer, 2005). But fluctuation should not be too slow: when people are in a bad mood for months, we enter the realm of mood disorders, such as dysthymia or major depression (Davidson et al., 2002a,b).

### Mood and cognitive processing

Why should we see mood as adjusting the mind's mode of operation? One good reason is that mood is known to affect mental functioning in systematic ways, across a wide array of phenomena. Relative to a bad mood, for example, a good mood tends to widen the spotlight of visual attention (Rowe et al., 2007; Schmitz et al., 2009), reduce the attractiveness of familiar stimuli (de Vries et al., 2010), and decrease the perceived bioenergetics costs of certain actions (such as climbing a hill; Zadra and Clore, 2011). A good mood also tends to increase the scope and impact of routine memory-based thinking, evidenced in broader associative memory retrieval (Bolte et al., 2003; Rowe et al., 2007), stronger use of scripts in episodic memory retrieval (Bless et al., 1996), and stronger sensitivity to social stereotypes in person judgment (Park and Banaji, 2000). It can facilitate creative problem-solving (Baas et al., 2008; Subramaniam et al., 2008; Davis, 2009), and reduces the degree of conflict-driven control (Van Steenbergen et al., 2010). And, on the downside, a good mood can make people more sensitive to weak arguments in persuasion (Bless et al., 1990; Forgas, 2007), and to misleading information in eyewitness memory (Forgas et al., 2005).

Most theorists assume that these changes are functional, and that mood plays a useful role in the workings of the mind. According to the *affect-as-information* hypothesis (e.g., Clore and Huntsinger, 2007), mood provides experiential and bodily information about the value of whatever comes to mind, a free-floating “somatic marker” (Damasio, 1996) that signals whether your cognitions and actions are generally on the right track or not. A related proposal is that a good mood loosens the control of deliberate, analytic but effortful “System 2” processing, thereby increasing the impact of memory-based low-cost “System 1” operations (Bar, 2009; Kahneman, 2011). What these and other accounts have in common is that mood is viewed as adjusting or “tuning” the operational principles of our mind in ways that are, on average, believed to increase the adaptive value of our behavior.

This tuning is usually discussed in terms of two basic modes of operation, with a good mood promoting an associative, “broad big-picture” style of thinking that relies on heuristics, i.e., rules of thumb that tend to work well in many but not all cases, and a bad mood promoting a narrower, more conservative and careful focus on the details instead (see Fiedler, 2001; Clore and Huntsinger, 2007; Friedman and Förster, 2010; Zadra and Clore, 2011; Shiota and Kalat, 2012, for reviews). Loosely speaking, the idea is that a good mood tells you to trust your instincts and go out there to explore, whereas a bad mood leads you to be more wary, to mentally stay put, and check out the details of whatever current problem seems to be at hand. Importantly, both modes of operation have their uses: there are times when it is best to stay put and recover from fatigue, injury or the stress of the unknown, but we also occasionally need to go out there into the wild, if only to look for food, mates, or creative solutions that improve the quality of life. In a bio-energetic perspective (e.g., Zadra and Clore, 2011), mood is seen as directly *signaling* the amount of resources available for such exploratory behavior.

### Mood and language processing

So what about language processing? In view of the above, it seems unlikely that the operational principles of our language architecture would remain completely unaffected. And indeed, mood has been reported to affect such diverse things as the level of abstractness in verbal autobiographical narrative (Beukeboom and Semin, 2005), the unusualness of words generated in a verbal association task (Isen et al., 1985), the response to syntactic anomalies (Vissers et al., 2010), the response to mood-incongruent words (Chung et al., 1996; Kiefer et al., 2006; Pratt and Kelly, 2008) and to mood-incongruent developments in discourse (Egidi and Gerrig, 2009; Egidi and Nusbaum, 2012), and the response to unexpected neutral concepts in sentences or discourse (Federmeier et al., 2001; Chwilla et al., 2011; Lai et al., 2012; Pinheiro et al., 2013).

Here, we explore whether mood affects the anticipation of referents during discourse comprehension. More specifically, we look at the processing of phrases such as “Linda praised David because …,” and ask to what extent mood tunes the degree to which readers predict who will be talked about next. An important reason for asking this particular question is that establishing reference is a core aspect of language comprehension (Clark, 1996; Garrett and Harnish, 2007; Van Berkum et al., 2007). Reference is critical to understanding “who does what to whom” in language—operating at the interface between timeless sentence meaning and fully contextualized utterance meaning, reference links the generic linguistic code to specific entities in the discourse. Establishing reference (or “joint attention”) is also at the heart of the human “interaction engine,” the shared infrastructure for communication that underlies linguistic and non-linguistic communication alike (Levinson, 2006; Tomasello, 2008). Therefore, an examination of how mood affects this process has potentially broad implications, beyond the sentence given.

### The implicit causality bias

In the studies reported below, we build on earlier research involving verb-based *implicit causality*. Implicit causality information is stereotypical knowledge about the causal roles of people engaged in interpersonal events denoted by verbs like “praise” or “apologize” (Garvey and Caramazza, 1974). When asked to complete a fragment such as “Linda praised David because … ” in an off-line test, readers and listeners are inclined to continue with something about David, e.g., “… because he had done well.” However, after “Linda apologized to David because …,” people tend to continue with something about *Linda* instead. In “NP1 verb-ed NP2 because … ” constructions, specific interpersonal verbs like “praise” and “apologize” thus supply information about whose behavior or state is the more likely immediate cause of the event at hand (the person denoted by NP2 with “praise,” and by NP1 with “apologize”). This verb-specific asymmetry is referred to as the implicit causality bias, and verbs like “praise” and “apologize” are sometimes labeled NP2- and NP1-verbs.

It turns out, at least under conditions where the participants' mood was not manipulated, that this implicit causality bias can rapidly lead readers to anticipate, *on the fly*, who will be in focus in the subsequent because-clause. Evidence for this comes from studies that compared the processing of sentences in which the because-clause continues with a bias-consistent referring pronoun (as in “Linda praised David because he … ”) to that of sentences where the referring pronoun is bias-*in*consistent (as in “David praised Linda because he … ”). In self-paced reading and eye tracking studies (Koornneef and Van Berkum, 2006; Featherstone and Sturt, 2010; Pyykkönen and Järvikivi, 2010), bias-inconsistent pronouns caused readers to slow down right at or shortly after the critical pronoun. Comparable evidence for such anticipation of referents comes from an event-related brain potentials follow-up (Van Berkum et al., 2007), where the processing costs of bias-inconsistent pronouns emerged in ERPs right at the critical pronoun, as a widely distributed positive ERP effect between 400–700 ms. These behavioral and neurophysiological processing costs of bias-inconsistent pronouns show that readers were at that point in the sentence indeed anticipating a reference to somebody else.

In the below ERP experiment, we used the positive ERP deflection to bias-inconsistent pronouns observed by Van Berkum et al. (2007) as an index of the degree to which readers use implicit causality information to anticipate who will be talked about next, as the discourse unfolds. Based on mood research in other domains (e.g., script-based memory retrieval, interpersonal judgment; Bless et al., 1996; Park and Banaji, 2000), we suspected that a good mood might foster such referential anticipation, and a bad mood might attenuate it. After all, anticipation based on implicit causality can be seen as a form of heuristic thinking, where people use a rule of thumb to extrapolate from what they reliably know—for example, that some David praised some Linda, and that the speaker is about to give a reason for this event—to what else might soon be the case: that the speaker is going to talk about Linda. Combining this with the Van Berkum et al. (2007) finding, we predicted that positive mood induction should promote anticipation based on implicit causality, reflected in a large globally distributed differential ERP positivity to bias-inconsistent pronouns around 400–700 ms. In contrast, negative mood induction should lead to a weaker reliance on implicit causality, observable as a weaker, possibly even absent differential ERP positivity to bias-inconsistent pronouns.

We also included a control measure that involved a qualitatively different aspect of language processing: syntactic parsing. We probed the latter by means of subject-verb number agreement violations (as in “The girls is watching TV”), relative to a corresponding correct phrase structure (e.g., “The girls are watching TV”). Such syntactic agreement violations are known to reliably elicit a so-called P600 effect (Hagoort et al., 1993; Osterhout et al., 2012), sometimes preceded by an earlier anterior negativity. Reasoning from the idea that mood would particularly affect the use of conceptual rules of thumb (scripts, stereotypes, etc.), we did not specifically predict a mood effect on the readers' response to syntactic agreement violations.

## EEG experiment: methods

### Participants

The EEG-study was conducted with 55 right-handed female native speakers of Dutch without diagnosed neurological or reading impairment, and without current use of antidepressants. All participants gave informed consent, in accordance with the Declaration of Helsinki. Six participants were excluded because of technical or other problems in one or both of their recording sessions, and 17 more participants lost too many segments to EEG artifacts (see below), leaving 32 participants for analysis, with a mean age of 21.4 years (range 18–29).

We checked for signs of depression by means of a Dutch adaptation of the *Positive and Negative Affect Schedule* (PANAS, Watson et al., 1988; Peeters et al., 1996). Participants rated the frequency with which they generally experience each of 10 positive and 10 negative emotions on a 5-point scale ranging from “(almost) never” (1) to “very often” (5). The PANAS uses separate cumulative scores for the positive affect (PA) and negative affect (NA) subscales, with scores ranging from 10 to 50 for each. For our 32 respondents, mean PA was 34.2 (range 29–43) and mean NA was 21.3 (14–33), which, according to Dutch norming data (Peeters et al., 1996) are representative of the general non-clinical population. Also, none of the individual participants had extreme scores that might indicate depression (PA ≤ 26, NA ≥ 34).

Because of the nature of our mood manipulation (see below), we also collected participant scores on a Dutch adaptation of the *Interpersonal Reactivity Index* (IRI, Davis, 1983), with a particular focus on the first three of its four 7-item subscales: Fantasy (FS), the tendency to transpose oneself imaginatively into feelings and actions of fictitious characters in books, movies and plays, and, in one item, of oneself in the future (essentially a measure of narrative transportation, Green, 2004); Empathic Concern (EC), the “other-oriented” tendency to experience compassion and concern for other people; and Personal Distress (PD), the “self-oriented” tendency to have feelings of unease and discomfort in tense interpersonal settings. Participants rated items on a 5-point scale (0 = not typical for me; 4 = very typical for me), and scores were averaged per subscale. Mean IRI scores were 2.7 for FS (range 0.9–4.0), 2.8 for EC (1.6–4.0), and 1.8 for PD (0.7–3.3).

### Language materials

#### Implicit causality items

To assess referential anticipation based on implicit causality, we expanded the original Dutch item set of the Van Berkum et al. (2007) ERP study into 144 two-variant mini-stories using the same construction criteria and implicit causality verbs as used for that ERP study. Half of the stories used an NP1-biased implicit causality verb (e.g., “apologize”) in the critical sentence, leading readers to expect more information about the first-mentioned character. The other half used an NP2-biased implicit causality verb (e.g., “praise”), leading readers to expect more information about the second-mentioned character. The critical manipulation was whether the subsequent singular gender-marked personal pronoun confirmed or disconfirmed those expectations. Because the Dutch equivalent of singular “she” (“zij”) also allows for a plural gender-unmarked reading, we always used the Dutch equivalent of unambiguous singular “he” (“hij”), and made sure that the pronoun confirmed or disconfirmed expectations by switching the position of the male and female character in the same sentence. A translated example item is shown in (1), with the critical pronoun marked for expository purposes only; see the Appendix for more examples (also in Dutch).

(1a) Implicit causality: bias-consistent pronoun
  Joe Biden and Sarah Palin prepared for a very important debate. They were both nervous, as this debate would certainly affect the elections. Sarah feared Joe because **he** was fully aware of her ignorance.
(1b) Implicit causality: bias-inconsistent pronoun
  Joe Biden and Sarah Palin prepared for a very important debate. They were both nervous, as this debate would certainly affect the elections. Joe feared Sarah because **he** was fully aware of her popularity.

The strength of the implicit causality bias was checked in a paper-and-pencil pretest with 40 Dutch participants (32 female; mean age 21.7, range 18–27) who read our stories truncated right after “because …,” and were asked to complete them while using “he” or singular “she.” A story with 50% “he” and 50% “she” completions (across all respondents) is unbiased, whereas a story with 100% “he” and 0% “she” completions, or vice versa, is fully biased. All selected stories had a bias toward the intended male or female character of at least 70%, with a mean bias of 88%.

Of the 40 implicit causality verbs used in the original set, 22 have rather negative connotations (e.g., “torment,” “tease,” “loathe”; see Van Berkum et al., 2007), against 11 with positive connotations (e.g., “praise,” “admire”). To redress the balance, we eliminated two of the weakest negative verbs (“mislead,” “intimidate”), and used the remaining negative verbs less often than the others: across the 144 two-variant IC-critical stories, the 20 negative verbs were used 64 times (average 3.2, range 1–4 times, 44% of the set), the 11 positive verbs were used 47 times (average 4.3, range 3–6 times, 33% of the set), and the 7 neutral verbs were used 33 times (average 4.7, range 3–6 times, 23% of the set).

To mask the frequent use of “omdat hij” in critical sentences, we also used “omdat” followed by other pronouns earlier in the implicit causality stories (21 times) as well as in some of the separate syntax stories described below (14 times). Further distraction was provided by setting up comparable two-person scenarios *without* “omdat” in the separate syntax stories (34 times), by mixing definite NPs (e.g., the neighbor) with proper names of celebrities (e.g., Tom Cruise) and other persons in the implicit causality stories (55, 38, and 6% respectively), by mixing past and present tense (74 and 26% respectively), and by having highly varied materials in terms of contents and genre.

#### Syntax items

We created 144 additional stories or story fragments that contained a subject-verb agreement violation, or a correctly agreeing control verb, in sentence-medial position. A translated example item is shown in (2), with the critical verb marked for expository purposes only; see the Appendix for more examples.

(2a) Syntax: correct subject-verb agreement
  Paul and Jim really like to challenge each other all the time. The boys *turn* even the slightest difference of opinion into a bet.
(2b) Syntax: violated subject-verb agreement
  Paul and Jim really like to challenge each other all the time. The boys *turns* even the slightest difference of opinion into a bet.

To minimize session time and participant fatigue, only half of the required 144 syntax items were independent stories. The other syntax items were realized by adding a single coherent continuation sentence to bias-consistent implicit causality stories. Anomalous verbs could be singular or plural (55 vs. 45%), and were embedded in active present- or past-tense constructions in which the specific verb itself was relatively unpredictable. Anomalous and correct verbs were set-wise matched on average written word frequency (Twente Corpus log string-frequency of 3.3 and 3.2, respectively) and average word length (6.3 and 6.4 letters respectively, range 3–10). Critical words in the implicit causality and syntax subdesigns were matched on average position in the story (at 27.8 and 26.8 words, respectively). The resulting full item set consisted of 216 critical mini-stories of two, three, or four sentences with an average length of 37.3 words, as well as 24 filler items used for starting or resuming the task after a break. All Dutch items can be obtained from the first author.

Every participant saw half of the items in their first recording session (with either positive or negative mood induction) and the other half in their second recording session (with negative or positive mood induction). A single story item was presented to any one participant in just one of its two variants. Each of eight different lists contained 108 critical stories: 36 bias-inconsistent, 36 bias-consistent of which half syntactically anomalous and half syntactically correct, and 36 pure syntax items of which half syntactically anomalous and half syntactically correct. Four lists were generated by rotating conditions over a single base randomization using half of the items, four more were generated by using the other half of the items, and each of the resulting lists featured equally often in sessions 1 and 2, as well as, fully crossed, with positive and negative mood induction. Item subsets used for condition rotation were matched on average bias strength (implicit causality items only), critical word length (syntax items only), and mean position of the critical word in the story. The item randomization procedure evenly distributed item types and conditions over five blocks, avoided lengthy sequences of bias-inconsistent and/or syntactically anomalous items (max 4 in a row), and always had each block start with 2–3 fillers. Each final list contained 120 stories, and was preceded by a 10-item practice block.

### Mood induction and manipulation check

We used short film clips to change mood in a positive or negative direction, as the effects of such clips are known to be robust and relatively long-lasting (Gross and Levenson, 1995; Rottenberg et al., 2007). For positive and negative mood induction, we selected five fragments from *Happy Feet* and *Sophie's Choice* respectively, movies that have been used effectively in earlier mood research. Fragments were chronologically selected such that they were maximally cheerful or depressing, relatively coherent as a unit, and comprehensible in terms of the larger plot unfolding over these clips. Fragments lasted for some 3.7 min (range 2:58–5:23 m:ss), so that participants saw about 18–19 min of cheerful or depressing film per session. Post-session film ratings in which participants evaluated the clips they had seen on two positive mood-related adjectives (*cheerful* and *funny*) and two negative mood-related adjectives (*sad* and *moving*), mixed with six less clearly valenced adjectives (such as *intense* or *complicated*) confirmed the valence of our selections, with Happy Feet consistently rated as positive, and Sophie's Choice rated as negative, by all participants (see also Table 1, left panel, in the Results section).

**Table 1:** **Post-session film rating means (bold) and standard deviations (regular) for Happy Feet and Sophie's Choice clips seen in the EEG experiment (left) or the behavioral post-test (right), on a 7-point adjective rating scale (1 = not at all, 7 = very)**.

**Film rating (1 = not at all, 7 = very)**	**EEG experiment**				**Behavioral experiment**			
	**Happy Feet**	**Sophie's Choice**	***F*_(1, 30)_**	***p***	**Happy Feet**	**Sophie's Choice**	***F*_(1, 38)_**	***p***
Cheerful (“vrolijk”)	**6.13**	**1.50**	1173.4	000^***^	**5.95**	**1.50**	342.8	000^***^
	0.6	0.6			0.9	0.6		
Funny (“grappig”)	**5.75**	**1.47**	608.1	000^***^	**5.65**	**1.30**	429.3	000^***^
	0.8	0.7			0.8	0.5		
Sad (“zielig”)	**3.23**	**5.75**	131.4	000^***^	**2.90**	**6.15**	94.8	000^***^
	1.3	0.8			1.4	0.5		
Moving (“aangrijpend”)	**2.47**	**5.88**	246.5	000^***^	**3.70**	**6.35**	59.6	000^***^
	1.3	0.7			1.4	0.7		
Intense (“heftig”)	**1.69**	**5.59**	458.7	000^***^	**1.80**	**5.80**	158.3	000^***^
	0.6	1.0			1.0	1.0		
Complicated (“ingewikkeld”)	**1.56**	**3.31**	58.8	000^***^	**1.35**	**3.60**	57.7	000^***^
	0.6	1.3			0.6	1.2		
Beautiful (“mooi”)	**4.75**	**4.81**	0.0	828	**5.20**	**4.85**	1.0	319
	0.9	1.2			1.0	1.2		
Interesting (“interessant”)	**4.13**	**5.44**	26.3	000^***^	**4.30**	**5.75**	17.4	000^***^
	1.0	0.9			1.3	0.9		
Exciting (“spannend”)	**2.97**	**4.57**	48.2	000^***^	**2.80**	**5.10**	31.9	000^***^
	1.2	1.0			1.5	1.0		
Boring (“saai”)	**1.75**	**2.26**	3.7	063	**1.85**	**1.80**	0.0	854
	0.8	1.2			1.0	0.7		

*F-tests reflect differences in rating of Happy Feet and Sophie's Choice clips, analyzed within-participants in the EEG study and between-participants in the behavioral experiment*.

*** *p < 0.001*.

To assess the mood of our participants before and throughout each session, we adapted a paper-and-pencil self-report questionnaire used in earlier mood research (de Vries, 2008). Participants were asked to quickly indicate how they felt “at this time” (for the pre-experiment measurement) or “during the preceding reading block,” using 25 common state adjectives and a 7 point scale ranging from 1 = *totally not* to 7 = *very much*. Mood was quantified as the average score across ten strongly valenced state adjectives: Dutch *vrolijk*, *tevreden*, *opgewekt*, *positief*, *goed*, and *negatief*, *verdrietig*, *somber*, *down*, *slecht* (approximate translations: *cheerful*, *content*, *good-humored*, *positive*, *good*, and *negative*, *sad*, *gloomy*, *down*, *bad*), after recoding all scales to a single dimension running from −3 (negative) to 3 (positive). Cronbach's alpha of this 10-item scale was 0.91, and the items were interspersed with 15 other state adjectives (Dutch *relaxed, zenuwachtig, actief, ontspannen, geconcentreerd, afgeleid, geïrriteerd, vermoeid, gemotiveerd, nieuwsgierig, geïnteresseerd, bang, onzeker, zelfverzekerd*, and *ongemakkelijk*; approximate translations: *relaxed, nervous, active, relaxed, focused, distracted, irritated, tired, motivated, curious, interested, scared, insecure, self-assured*, and *uncomfortable*), partly to avoid a focus on strongly valenced moods.

### Procedure

Participants were contacted and screened via email, and filled out the PANAS and IRI as part of that. They then came to the lab twice to take part in “an EEG experiment in which they would be performing two alternating tasks (watching short movie fragments and reading short texts), aimed to find out how well women can combine these two tasks and whether that depends on the type of movie they are actually watching.” Sessions were approximately 1 week apart and always took place at the same time of the day, with half of the participants having the positive mood induction first, and the other half having the negative mood induction first.

In each session, participants were comfortably seated in a normally lit room where, after electrode application, instructions, and a 10-story practice session, they first filled out the brief self-report questionnaire. Then, for each of 5 blocks, participants saw a film clip, then read 24 stories, subsequently filled out the self-report questionnaire again, and then had a short pause, until they felt ready for the next block. Participants were asked to focus on the story laid out by the five film clips, and to relax and avoid strong movement during those clips “for EEG measurement.” They were also asked to read the texts for comprehension (while avoiding blinks), and to ignore occasional errors. After reading a text, presentation of the next one could be initiated by the participant via a button-press. After the fifth block, participants filled out a brief 10-item film-rating questionnaire, and had a short post-session interview with the experimenter (with final debriefing after session 2). A single block lasted about 15 min, and an entire session lasted about 2.5–3 h.

Stories were presented in black 30 point Tahoma font on a white background on a fast TFT display positioned at about 80 cm distance. Each trial started with a 1000-ms “^***^” warning signal followed by a 500-ms centered fixation cross, after which the stories were presented word by word at the same position. As in the 2007 study, we used a variable serial visual presentation procedure in which the duration of each word depended on length and sentence position, using parameters based on natural reading times, a subjective assessment of the naturalness of the resulting presentation, and constraints imposed by the 75 Hz video refresh rate. Non-critical word duration consisted of a standard offset of 187 ms, and an additional 27 ms per letter (with an upper bound of 8 letters for each word; range 241–403 ms), clause-final words with a comma or period sign were prolonged by an additional 200 or 293 ms, respectively, and the screen went blank for 106 ms between words and for a 1000 ms between sentences. To avoid spurious ERP effects due to accidental differences in average word length across conditions (and, hence, shifted offset and onset potentials), the critical word, the preceding word and the two words following it were presented with a fixed duration of 318 ms, based on the average critical word length across all stories. Participants did not notice the alternation between completely variable and semi-fixed word duration presentation.

### EEG-recording, preprocessing, and analysis

EEG was recorded from 34 ActiCap active electrodes (BrainProducts, Germany) at standard 10–20 positions in an elastic cap, each referenced to the left mastoid, and with impedances kept below 20 kΩ. Signals were amplified with BrainAmps DC amplifiers (0.1–100 Hz band-pass), digitized at 500 Hz, re-referenced off-line to the mastoid average, and low-pass filtered at 35 Hz (24 dB/oct). Additional bipolar hEOG and vEOG signals were computed from F9-F10 and from Fp1-V1 (an additional electrode below the left eye) respectively. Segments ranging from 200 ms before to 1000 ms after critical word onset were extracted and baseline-corrected to a 200-ms pre-onset baseline. Segments with EEG or EOG signals exceeding ±75 μ V, a voltage step of over 50 μ V per ms, or extremely low activity (maximum amplitude difference below 0.5 μ V for at least 100 ms) were rejected, and the data of 17 participants with over 50% segment loss, on 36 segments per condition, in any condition of any of their two recording sessions, were excluded. For the remaining 32 participants, average segment loss was 11.4% in positive mood sessions and 11.4%, in negative mood sessions, with no asymmetry across sentence type. Remaining segments were averaged per participant, mood induction (positive vs. negative), sentence type (implicit causality vs. syntax), critical word type (unexpected vs. expected, based on implicit causality bias or syntactic agreement), and 6-electrode scalp recording quadrant (left-anterior: F3, F7, F9, FC1, FC5, FT9; right-anterior: F4, F8, F10, FC2, FC6, FT10; left-posterior: CP1, CP5, P3, P7, O1, PO9; right-posterior: CP2, CP6, P4, P8, O2, PO10). Mean amplitude values were analyzed with repeated-measures mood × critical word type × hemisphere × anterior-posterior ANOVAs in consecutive 100-ms latency ranges, using Greenhouse–Geisser/Box's epsilon correction for univariate *F*-tests with two or more df.

## EEG experiment: results

### Film ratings

Post-experiment evaluations of clips drawn from Happy Feet and Sophie's Choice are displayed in Table 1 (left half, EEG experiment). As intended, Happy Feet attracted stronger positive (“cheerful,” “funny”) ratings than Sophie's Choice, and Sophie's Choice attracted stronger negative (“sad,” “moving,” “intense”) ratings than Happy Feet.

### Aggregated mood ratings

As displayed in Table 2 (left half, EEG experiment), participants reported a moderately good mood when they came into the lab, and comparably so in both sessions. Subsequent positive mood induction with Happy Feet clips caused them to maintain their good mood throughout the experiment, whereas negative mood induction with Sophie's Choice clips caused their mood to deteriorate in all blocks.

**Table 2:** **Self-reported mood score means (bold) and standard deviations (regular) before and during the EEG experiment (left) or the behavioral post-test (right), on a 7-point aggregated scale (−3, maximally negative; 0, neutral; +3, maximally positive)**.

**Global mood rating (−3 = negative, +3 = positive)**	**EEG experiment**				**Behavioral experiment**			
	**Happy Feet**	**Sophie's Choice**	***F*_(1, 30)_**	***p***	**Happy Feet**	**Sophie's Choice**	***F*_(1, 38)_**	***p***
Mood before induction	**1.63**	**1.69**	0.68	418	**1.77**	**1.81**	0.05	831
	0.57	0.57			0.68	0.64		
Mood at end of block 1	**1.76**	**0.94**	51.37	000^***^	**1.85**	**0.96**	8.69	005^**^
	0.54	0.96			0.70	1.16		
Mood at end of block 2	**1.69**	**0.99**	43.72	000^***^	**1.78**	**0.98**	7.92	008^**^
	0.60	0.94			0.68	1.07		
Mood at end of block 3	**1.59**	**1.23**	21.77	000^***^	**1.59**	**1.09**	2.91	096
	0.68	0.71			0.81	1.03		
Mood at end of block 4	**1.76**	**1.11**	54.25	000^***^	**1.89**	**1.12**	9.04	005^**^
	0.62	0.92			0.65	0.95		
Mood at end of block 5	**1.80**	**1.05**	87.95	000^***^	**1.85**	**1.04**	8.81	005^**^
	0.55	0.82			0.73	0.95		
Avg. mood across block 1–5	**1.72**	**1.06**	97.23	000^***^	**1.79**	**1.03**	8.13	007^**^
	0.55	0.79			0.67	0.98		

*F-tests reflect differences in ratings for sessions involving Happy Feet or Sophie's Choice clips*.

*** p < 0.001;

** p < 0.01.

### Other current state ratings

In sessions with Sophie's Choice clips, participants judged themselves to be somewhat less relaxed [SC: 4.96 vs. HF: 5.23, *F*_(1, 30)_ = 6.10, *p* = 0.019], slightly more nervous [SC: 1.65 vs. HF: 1.42, *F*_(1, 30)_ = 5.32, *p* = 0.028], marginally more scared [SC: 1.41 vs. HF: 1.22, *F*_(1, 30)_ = 3.95, *p* = 0.056] and marginally less self-assured [SC: 4.78 vs. HF: 4.96, *F*_(1, 30)_ = 3.10, *p* = 0.088]. No other differences were significant (all *p*-values > 0.100).

### EEG: early components elicited by critical words

To assess the impact of mood on early attention-sensitive visual stimulus processes indexed by the N1 and P2, we extracted mean amplitudes in the latency range of 100–130 ms for the N1, and 160–190 ms for the P2, with latency ranges centered around N1 and P2 peak amplitudes at Pz and Oz, and across all critical words. Mood did not affect either component [N1: *F*_(1, 31)_ = 0.03, *MSE* = 77.44, *p* = 0.869, and P2: *F*_(1, 31)_ = 0.73, *MSE* = 47.84, *p* = 0.399, tested across 19 posterior electrodes; N1: *F*_(1, 31)_ = 0.13, *MSE* = 39.40, *p* = 0.722, and P2: *F*_(1, 31)_ = 1.27, *MSE* = 72.11, *p* = 0.268, tested across all electrodes].

### EEG: implicit causality items

**Figure 1:**
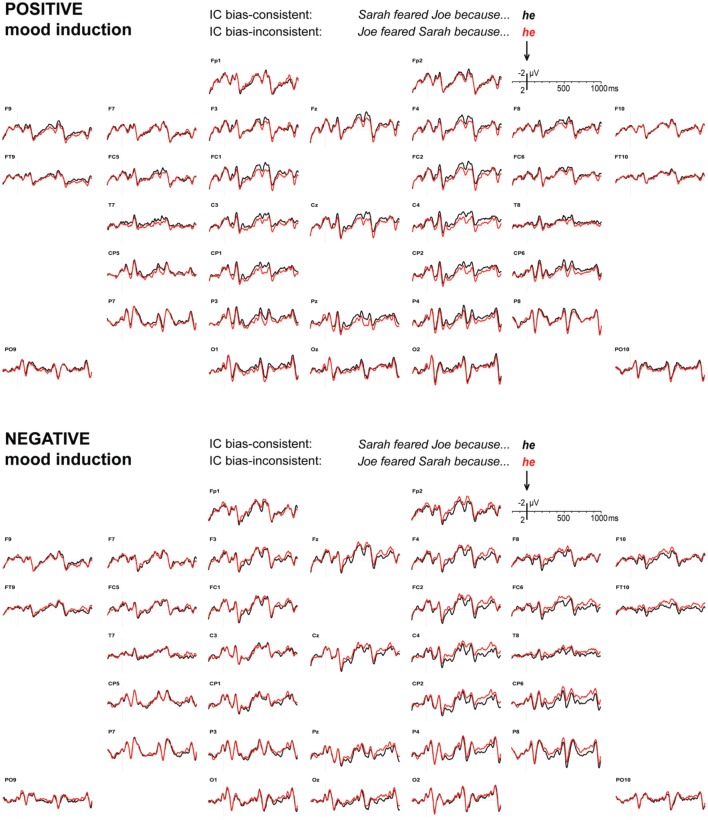
**Implicit causality.** ERPs to pronouns that are consistent (black) or inconsistent (red) with the implicit causality bias of a preceding verb, after positive **(top)** or negative **(bottom)** mood induction. Signals are arranged quasi-topographically, with negative voltage up and onset of the pronoun at 0 ms.

Figure 1 displays the grand average ERP waveforms to pronouns that are consistent or inconsistent with the verb-based implicit causality bias, after positive and negative mood induction. We had predicted a widely distributed ERP positivity to bias-inconsistent pronouns after positive mood induction, at least around 400–700 ms, relative to bias-consistent pronouns. The waveforms in Figure 1's top panel and the associated statistics confirm this prediction. For positive mood induction sessions, mean-amplitude quadrant ANOVAs revealed a small but reliable bias inconsistency effect in the latency ranges of 400–500 ms [*F*_(1, 31)_ = 5.34, *MSE* = 4.19, *p* = 0.028], and 500–600 ms [*F*_(1, 31)_ = 8.53, *MSE* = 4.81, *p* = 0.006] only. Neither of these effects differed as a function of anteriority, hemisphere, or the two considered together (all *p*-values > 0.600). Marginal bias inconsistency effects with *p*-values < 0.100 were found in the latency ranges of 100–200 ms [*F*_(1, 31)_ = 3.50, *MSE* = 4.07, *p* = 0.071], and 900–1000 ms [*F*_(1, 31)_ = 3.89, *MSE* = 4.64, *p* = 0.058]; no other 100-ms latency range revealed a reliable inconsistency effect (all *p*-values > 0.100).

Importantly, current ideas about how negative mood affects the use of heuristics led us to predict that negative mood induction would attenuate or perhaps even eliminate the positivity elicited by bias-inconsistent pronouns. As can be seen in Figure 1's bottom panel, this is indeed what we observed. In those latency ranges where the bias inconsistency effect had emerged under positive mood induction (400–500 and 500–600 ms), *negative* mood induction led to a reliable attenuation of the inconsistency effect [mood by consistency interaction, 400–500 ms: *F*_(1, 31)_ = 6.19, *MSE* = 3.91, *p* = 0.018, and 500–600 ms: *F*_(1, 31)_ = 13.81, *MSE* = 4.59, *p* = 0.001]. Furthermore, under negative mood induction, simple main effects did not reveal a reliable inconsistency-induced positive ERP deflection in these latency ranges [400–500 ms: *F*_(1, 31)_ = 0.92, *MSE* = 5.38, *p* = 0.346; 500–600 ms: *F*_(1, 31)_ = 3.83, *MSE* = 6.16, *p* = 0.059; the latter trend involves a negative deflection]^1^.

Under negative mood induction, bias-inconsistent pronouns elicited an unexpected negativity in the latency ranges of 200–300 ms [*F*_(1, 31)_ = 6.39, *MSE* = 2.40, *p* = 0.017], and 300–400 ms [*F*_(1, 31)_ = 5.13, *MSE* = 3.03, *p* = 0.031], larger over the right than over the left hemisphere [200–300: *F*_(1, 31)_ = 4.19, *MSE* = 0.62, *p* = 0.049; 300–400: *F*_(1, 31)_ = 3.06, *MSE* = 0.56, *p* = 0.090]. ERP effects in these latency ranges did not differ as a function of anteriority, or anteriority and hemisphere considered together (all *p-values* > 0.200). No other 100-ms latency range revealed a reliable inconsistency main effect (all *p*-values > 0.100), but subsequent hemisphere by inconsistency interactions, in the latency ranges of 400–500 ms [*F*_(1, 31)_ = 12.70, *MSE* = 0.50, *p* = 0.001], 500–600 ms [*F*_(1, 31)_ = 19.79, *MSE* = 0.37, *p* < 0.001], 600–700 ms [*F*_(1, 31)_ = 7.25, *MSE* = 0.59, *p* = 0.011], 800–900ms [*F*_(1, 31)_ = 6.01, *MSE* = 0.59, *p* = 0.020], and 900–1000 ms [*F*_(1, 31)_ = 10.09, *MSE* = 0.39, *p* = 0.003], do reveal a hemispheric asymmetry in the ERP effect elicited by bias-inconsistency.

### EEG: syntax items

**Figure 2:**
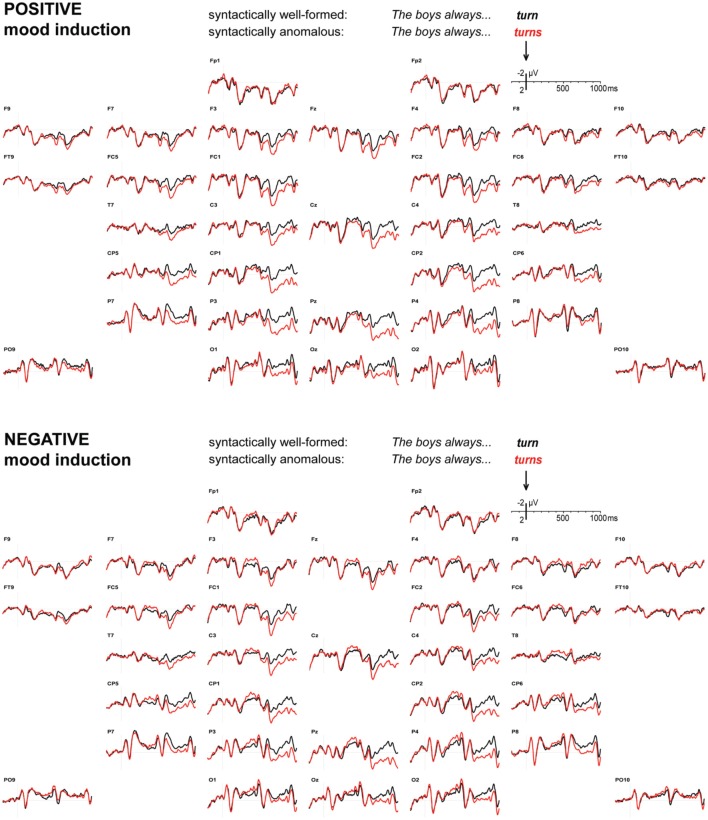
**Syntax.** ERPs to syntactically well-formed (black) or anomalous (red) verbs, after positive **(top)** or negative **(bottom)** mood induction. Onset of the verb at 0 ms, other graphics details as in Figure 1.

Figure 2 displays the grand average ERP waveforms to verbs that respected or violated a syntactic subject-verb agreement rule, after positive and negative mood induction. Syntactic violations elicited a widely distributed but posteriorly dominant P600 effect in both mood conditions, with a (descriptively) slightly later onset in the negative mood condition. In line with this, a combined analysis of variance revealed reliable main effects of syntactic anomaly in 600–700 ms [*F*_1,31_ = 16.55, *MSE* = 9.97, *p* < 0.001], 700–800 ms [*F*_1,31_ = 20.67, *MSE* = 7.27, *p* < 0.001], 800–900 ms [*F*_1,31_ = 19.17, *MSE* = 8.31, *p* < 0.001], and 900–1000 ms [*F*_1,31_ = 19.25, *MSE* = 7.41, *p* < 0.001]. No main effect of syntactic anomaly emerged in the 500–600 ms latency range [*F*_(1, 31)_ = 2.51, *MSE* = 9.16, *p* = 0.123], nor in any of the earlier latency ranges (all *p*-values > 0.300).

**Figure 3:**
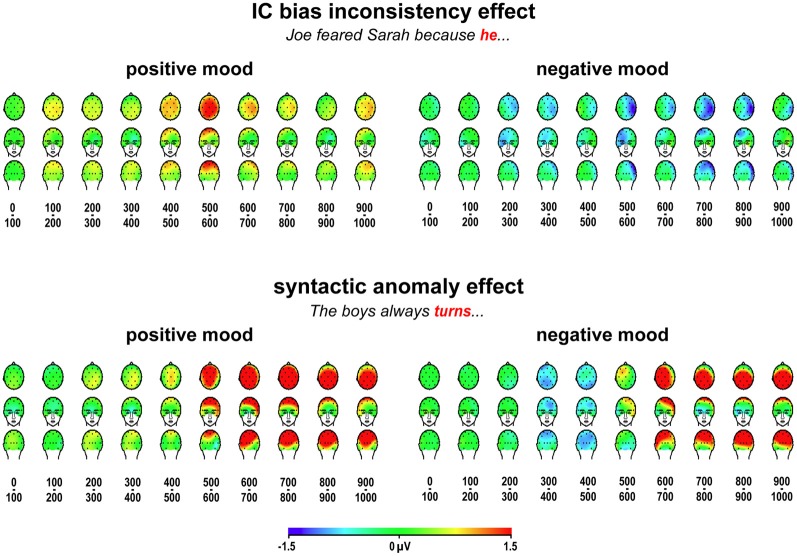
**ERP effects to implicit causality bias inconsistency and syntactic anomaly.** Scalp distributions of grand average differential ERP effects to bias-inconsistent (vs. -consistent) pronouns, and to syntactically anomalous (vs. correct) verbs, for ten 100-ms latency ranges after critical word onset, in positive and negative mood induction sessions.

Importantly, mood induction did not reliably affect the size of the P600 anomaly effect in any of these P600 effect latency ranges [500–600 ms: *F*_(1, 31)_ = 2.31, *MSE* = 7.23, *p* = 0.139, 600–700 ms: *F*_(1, 31)_ = 0.73, *MSE* = 8.80, *p* = 0.398, 700–800 ms: *F*_(1, 31)_ = 1.93, *MSE* = 8.20, *p* = 0.175, 800–900 ms: *F*_(1, 31)_ = 1.03, *MSE* = 4.89, *p* = 0.318, and 900–1000 ms: *F*_(1, 31)_ = 0.09, *MSE* = 6.90, *p* = 0.764]. Pooling mean amplitudes across the entire 500–1000 ms latency range also did not lead to a significant P600 effect size difference [mood by anomaly: *F*_(1, 31)_ = 1.33, *MSE* = 5.59, *p* = 0.257], and merely resulted in a highly reliable main effect [*F*_(1, 31)_ = 19.10, *MSE* = 6.21, *p* < 0.001]. Furthermore, mood did not affect the laterality of the P600 effect in any of the relevant latency ranges (all *p-values* > 0.300), nor did it affect its distribution over the anterior-posterior axis (all *p-values* > 0.400). Minor distributional differences (see Figure 3) gave rise to reliable mood by anomaly by hemisphere by posteriority interactions in the 500–600 ms latency range [*F*_(1, 31)_ = 4.65, *MSE* = 0.27, *p* = 0.039] and the 800–900 ms latency range [*F*_(1, 31)_ = 4.43, *MSE* = 0.24, *p* = 0.044], as well as to a marginal interaction in the 700–800 ms latency range [*F*_(1, 31)_ = 3.27, *MSE* = 0.20, *p* = 0.080; all *p*-values > 0.100 in the other latency ranges].

Mood induction did interact with syntactic anomaly in two earlier latency ranges: 300–400 ms [*F*_(1, 31)_ = 6.10, *MSE* = 4.34, *p* = 0.019], and 400–500 ms [*F*_(1, 31)_ = 6.79, *MSE* = 4.86, *p* = 0.014]. This can be traced to small *negative* syntactic anomaly effects in these latency ranges [300–400 ms: *F*_(1, 31)_ = 7.11, *MSE* = 3.30, *p* = 0.012; 400–500 ms: *F*_(1, 31)_ = 6.01, *MSE* = 5.25, *p* = 0.020] under negative mood induction, but not under positive mood induction [300–400 ms: *F*_(1, 31)_ = 0.91, *MSE* = 6.50, *p* = 0.348; 400–500 ms: *F*_(1, 31)_ = 1.06, *MSE* = 5.93, *p* = 0.310].

Figure 3 displays scalp distributions of the observed effects to bias-inconsistent pronouns and syntactic agreement violations, and can serve as an interim summary of our findings. In line with our predictions, we replicate the Van Berkum et al. (2007) result, a widely distributed positivity around 400–700 ms to bias-inconsistent pronouns, in positive mood sessions, and we see no such positivity to bias-inconsistent pronouns in negative mood sessions. Also in line with our predictions, we see a widely distributed large P600 effect to syntactic anomalies in both mood conditions. In negative mood sessions, we also obtain two additional effects that we did not predict: a right-dominant negativity to bias-inconsistent pronouns first emerging around 200–400 ms, and a more symmetrical, posteriorly dominant negativity to syntactic anomalies around 300–500 ms.

### EEG: participants with sufficient moodshift

Before interpreting the above, we need to consider the fact that our movie clip manipulation did not affect all participants equally effectively. To explore this, we separately examined the data for participants whose self-reported mood after positive vs. negative mood induction, quantified on the overall 10-item mood scale and pooled across 5 experimental blocks, differed by at least half a scale point in the right direction. In the resulting group (*N* = 16, of which 7 with positive, and 9 with negative mood induction first), average mood score differed by an average 1.19 scale points (1.76 vs. 0.57 for positive vs. negative mood induction) [*F*_(1, 15)_ = 116.76, *p* < 0.001].

**Figure 4:**
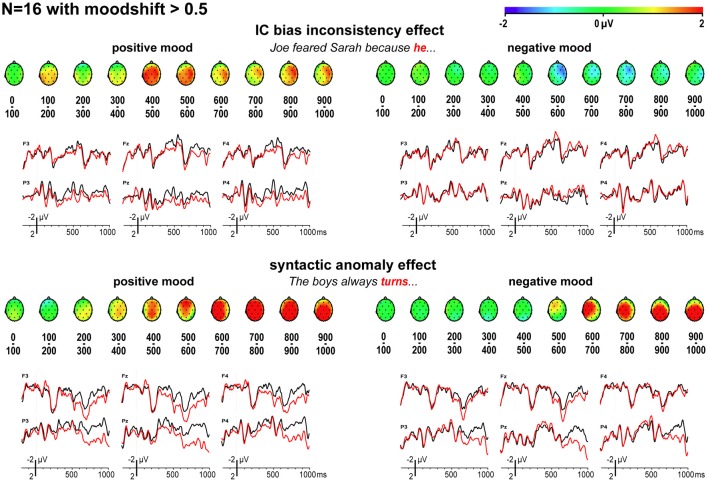
**ERP effects for implicit causality and syntax, for mood-responsive participants only.** Scalp distributions of differential ERP effects to bias-inconsistent (vs. -consistent) pronouns and syntactically anomalous (vs. correct) verbs, and associated ERPs, for participants whose self-reported mood shifted at least half a scale point in the intended direction.

As shown in Figure 4, for those participants where the mood manipulation worked as intended, the ERP findings for implicit causality are as predicted: a reliable globally distributed ERP positivity to bias-inconsistent pronouns in the latency ranges of 400–500 ms [*F*_(1, 15)_ = 8.73, *MSE* = 4.44, *p* = 0.010] and 500–600 ms [*F*_(1, 15)_ = 7.25, *MSE* = 4.08, *p* = 0.017] after positive mood induction, no such ERP positivity [400–500 ms: *F*_(1, 15)_ = 0.01, *MSE* = 3.97, *p* = 0.921; 500–600 ms: *F*_(1, 15)_ = 2.07, *MSE* = 8.38, *p* = 0.171] after negative mood induction (nor any other reliable ERP effects in any other latency range, all *p*-values > 0.100), and reliably different inconsistency effects in these two moods [mood by inconsistency interaction, 400–500 ms: *F*_(1, 15)_ = 9.52, *MSE* = 2.17, *p* = 0.008; 500–600 ms: *F*_(1, 15)_ = 13.10, *MSE* = 3.52, *p* = 0.003]. These are *exactly* the findings one would expect if a positive mood leads people to use implicit causality information to anticipate upcoming referents, and if a negative mood abolishes such heuristic anticipation.

Furthermore, mood had only a limited effect on the P600 effect elicited by syntactic anomalies. A reliable positivity emerged as a main effect across mood in the latency ranges of 500–600 ms and beyond (all *p*-values < 0.050), with the size of this P600 effect not depending on mood in any of these latency ranges (all *p-values* > 0.200), but with two mood by anomaly interactions in the 300–400 and 400–500 ms latency range (*p* = 0.047 and 0.031, respectively), reflecting the visible earlier onset of the positivity for those in a positive mood. The N1 and P2 components were not affected by mood [N1: *F*_(1, 15)_ = 0.02, *MSE* = 20.14, *p* = 0.902, and P2: *F*_(1, 15)_ = 0.00, *MSE* = 44.25, *p* = 0.985, across 19 posterior electrodes; N1: *F*_(1, 15)_ = 0.05, *MSE* = 45.17, *p* = 0.834, and P2: *F*_(1, 15)_ = 0.00, *MSE* = 78.20, *p* = 0.989, across all electrodes].

**Figure 5:**
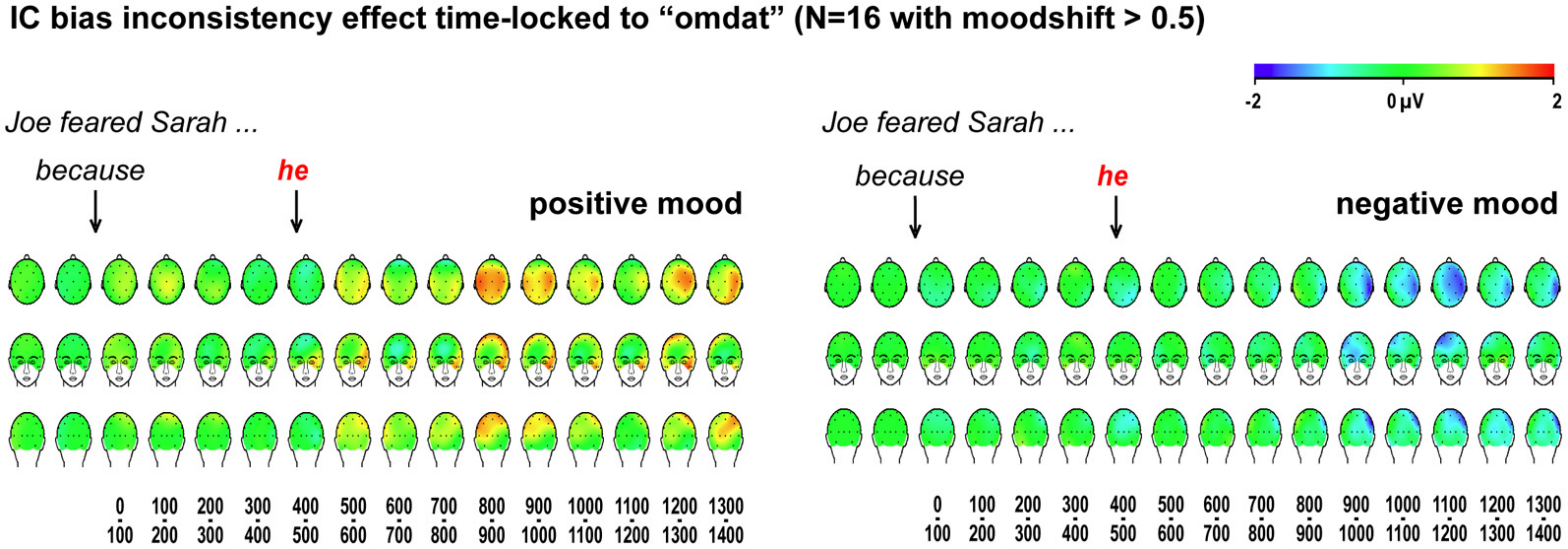
**ERP effects for implicit causality items, time-locked to the pre-critical word.** Scalp distributions of differential ERP effects to bias-inconsistent (vs. -consistent) pronouns, in 100-ms latency ranges, time-locked and baseline-corrected to the preceding word “omdat,” for participants whose self-reported mood shifted at least half a scale point.

The critical waveforms in Figure 4, obtained with implicit causality under a positive mood, begin to differentiate early, with traces of a posterior positivity already in the 100–200 ms latency range. Although not reliably different in the statistics, such early differential trends could signal a problem with ERP baseline correction, caused by differential effects to the preceding word. We examined this possibility by analyzing ERPs elicited by the preceding word “omdat” (“because”), presented 428 ms before the critical pronoun. As visible in Figure 5, our critical effects are clearly tied to the differential status of the subsequent pronoun—no differential effects were elicited by “omdat” in the two relevant item conditions, in either mood.

It is important to understand why only half of our 32 participants displayed a sufficient change in mood upon viewing Sophie's Choice vs. Happy Feet clips, and why the rest did not. Part of the variability in how our manipulation affected the participants might have come about because of accidental differences in pre-experiment mood. At the start of their positive or negative mood induction sessions (so before seeing Happy Feet or Sophie's Choice later in the session), the mood-responsive group did not differ on 10-item baseline mood [before HF: 1.75, before SC: 1.63, *F*_(1, 15)_ = 0.85, *p* = 0.372, 10-item scale]. In the remaining group, however, participants arrived with a reliably better mood at sessions where they would see Sophie's Choice than at sessions where they would see Happy Feet [before HF: 1.50, before SC: 1.76, *F*_(1, 15)_ = 5.80, *p* = 0.029]. This will to some extent have counteracted the manipulation.

Furthermore, an examination of the 15 self-report state parameters *not* captured in the 10-item overall mood scale revealed a qualitatively different pattern of self-reported state changes in these two groups. In the mood-responsive group, presenting Sophie's Choice instead of Happy Feet had a substantial effect on many parameters: these participants reported feeling less relaxed [probed via two items, “relaxed”: *F*_(1, 15)_ = 12.02, *p* = 0.003; “ontspannen”: *F*_(1, 15)_ = 17.58, *p* = 0.001], less focused [*F*_(1, 15)_ = 5.28, *p* = 0.036], less motivated [*F*_(1, 15)_ = 7.76, *p* = 0.014], less self-assured [*F*_(1, 15)_ = 6.62, *p* = 0.021], more nervous [*F*_(1, 15)_ = 8.78, *p* = 0.010], more afraid [*F*_(1, 15)_ = 5.22, *p* = 0.037], marginally more uncomfortable [*F*_(1, 15) = 3.69_, *p* = 0.074], and marginally less active [*F*_(1, 15)_ = 3.54, *p* = 0.079]. In the remaining group, however, presenting Sophie's Choice instead of Happy Feet did not reliably affect any of the 15 state parameters, apart from a slight *de*crease in irritation [*F*_(1, 15)_ = 4.39, *p* = 0.054; all other *p*-values > 0.100]. If anything, this suggests that Happy Feet, our intended positive mood inducer, actually irritated participants in this group, at least more so than Sophie's Choice. Furthermore, in this group, the two movies had no other detectable effects on self-reportable states.

What might have caused these differences in response style? The two groups did not reliably differ on age [*F*_(1, 31)_ = 0.28, *p* = 0.600], positive PANAS score [*F*_(1, 31)_ = 0.25, *p* = 0.624], negative PANAS score [*F*_(1, 31)_ = 1.10, *p* = 0.303], or the IRI subscales EC [*F*_(1, 31)_ = 0.72, *p* = 0.403] and PD [*F*_(1, 31)_ = 0.73, *p* = 0.788]. However, one difference between the groups approached significance: people in the mood-responsive group had marginally higher scores on the IRI Fantasy scale than those in the remaining group [20.7 vs. 17.7; *F*_(1, 31)_ = 3.60, *p* = 0.068]. Also, across all 32 participants, IRI Fantasy scores correlated significantly with self-reported mood after having seen Sophie's Choice (*r* = 0.40, *p* = 0.025), but *not* after having seen Happy Feet (*r* = −0.16, *p* = 0.386). It appears, therefore, that those participants who, as part of their general self-reported response style, tend to more strongly feel for, or feel along with, fictional characters in movies, novels, and plays were more sensitive to clips from Sophie's Choice than participants who score low on the IRI Fantasy scale. Higher IRI Fantasy scores in the mood-responsive group may well be part of the reason why Sophie's Choice worked as intended, inducing a large overall mood shift on the 10-item aggregate scale, as well as a large number of other state changes that are consistent with the intended effect of this movie (such as feeling less relaxed and more nervous). Conversely, lower IRI Fantasy scores in the remaining group may well be part of the reason why Sophie's Choice did not have the intended effect there, and may actually have had uncontrolled other effects, such as feeling slightly less irritated over Sophie's Choice relative to Happy Feet, instead of feeling worse.

## Summary

In all, the results of the EEG study can be summarized as follows. Self-report measures indicate that the movie-based mood manipulation is not equally effective for all 32 participants, presumably in part because of individual differences in narrative transportation (Green, 2004), the degree to which people feel for, or feel along with, characters in Sophie's Choice. But participants whose self-report measures indicate a reliable deterioration of mood after having seen Sophie's Choice, relative to having seen Happy Feet, display the ERP results we had predicted. In particular, the relevant ERP traces suggest that participants used implicit causality information associated with verbs like “praise” and “apologize” to anticipate who would initially be referred to in a subsequent clause when they were in a good mood, but not (or at least not noticeably so) when they were in a bad mood. Furthermore, in the same participants, a deterioration of mood delayed their response to syntactic agreement violations to some extent, but it did not in the end lead to a substantially smaller P600 effect. In all, our data suggest that under the conditions tested here, a bad mood selectively eliminates the heuristic anticipation of referents, while leaving subject-verb number agreement processing more or less intact.

## Story completion experiment

This result raises an interesting issue. Are readers in a bad mood *generally* less biased when reading implicit causality verbs? If so, then one would expect them to also give less biased completions of, say, “David praised Linda because … ” in a story completion test. Alternatively, perhaps a bad mood affects some *real-time* aspect of language comprehension, such as the ability or inclination to bring the implicit causality bias to bear sufficiently rapidly to predict who will be referred to right after “because,” as the sentence is unfolding. We suspected the latter to be more likely, and conducted a behavioral follow-up experiment to decide the issue. We used the same mood induction, but we truncated our implicit causality items after “because,” and asked new participants to complete those stories while using Dutch “he” or “she,” so that we could compute the bias supplied by the verb in those conditions. If a negative mood causes readers to perceive these verbs as less biased, the average verb bias should be lower than the bias observed with a positive mood, and also less than the 88% average bias observed in our mood-neutral pretest (or the 90% observed in the mood-neutral pretest associated with the Van Berkum et al., 2007 findings). Alternatively, if the mean bias ratings end up around the earlier 88–90%, independent of mood, we can infer that the attenuated ERP response to bias-inconsistent pronouns observed with a negative mood is not the result of a weaker bias as such.

### Participants

The behavioral experiment was conducted with 40 right-handed female native speakers of Dutch who all met the same participant selection criteria as used for the EEG experiment, and who all gave informed consent in accordance with the Declaration of Helsinki. For practical reasons, mood induction was manipulated as a between-participants factor, with random assignment of participants to either condition. The two groups of 20 participants in positive vs. negative mood induction were comparable on mean age (21.6 vs. 21.0 years; ranges 18–30 and 18–25), mean positive PANAS (33.2 vs. 34.5; ranges 27–41 and 28–44) and mean negative PANAS score (18.7 vs. 18.8; ranges 14–31 and 11–31), mean IRI FS score (2.7 vs. 2.5, ranges 1.1–3.7 and 1.6–3.4), IRI EC score (2.7 vs. 2.6, ranges 2.1–4.0 and 1.3–3.7), and IRI PD score (1.6 vs. 1.8, ranges 0.7–2.1 and 0.7–3.0).

### Materials and procedure

At stake was the off-line processing of implicit causality in a positive and negative mood. We therefore only used the original 144 three-sentence mini-stories constructed for that part of the design, and disregarded the agreement violation materials. All stories were truncated after (the Dutch equivalent of) “because”, and presented in its two character order variants, to different participants always:

(3) Joe Biden and Sarah Palin prepared for a very important debate. They were both nervous, as this debate would certainly affect the elections. Sarah feared Joe [Joe feared Sarah] because …

Stories were presented in the same order as in the EEG study, reusing the original randomizations while stripping them of all agreement-oriented items. Apart from the presence of a response task and the absence of EEG-related recording, the entire procedure (including mood induction, self-report, and trial timing) was identical to that in the EEG study. Also, stories were presented in the same manner as before, using identical word durations. The final word “omdat” (“because”) remained on the screen until participants began typing, and all typed responses were recorded verbatim on file. As in the original materials pretest, instructions asked participants to read each story attentively but to then immediately complete it with the first short completion that came to mind, using (Dutch equivalents of) “he” or “she,” the latter intended as singular. Typed responses were automatically coded as consistent or inconsistent with the implicit causality verb bias. A single block lasted about 15–20 min, and an entire session lasted about 2–2.5 h.

### Film and mood ratings

As in the EEG study, Happy Feet attracted stronger positive (“cheerful,” “funny”) ratings than Sophie's Choice, and Sophie's Choice attracted stronger negative (“sad”) ratings than Happy Feet, see Table 1 (right half, behavioral experiment). Also, as displayed in Table 2 (right half, behavioral experiment), the impact of mood induction was like that in the EEG study: participants reported a moderately good mood when they came into the lab, subsequent positive mood induction with Happy Feet clips caused them to maintain their good mood throughout the experiment, and negative mood induction with Sophie's Choice clips caused their mood to deteriorate in all blocks. In addition, those who had seen Sophie's Choice clips judged themselves to feel a little more uncomfortable [SC: 2.31 vs. HF: 1.47, *F*_(1, 38)_ = 6.91, *p* = 0.012], marginally more nervous [SC: 1.69 vs. HF: 1.31, *F*_(1, 38)_ = 3.06, *p* = 0.088] and marginally less relaxed [SC: 4.86 vs. HF: 5.38, *F*_(1, 38)_ = 3.52, *p* = 0.068]. No other differences were significant (all *p*-values > 0.100).

### Story completions

Participants in both groups produced equally biased story completions, with on average 90% (SD 4%, range 80–96%) bias-consistent story completions in the positive mood induction condition, and 91% (SD 4%, range 82–97%) in the negative mood induction condition [*F*_(1, 38)_ = 0.41, *MSE* = 19.00, *p* = 0.525].

### Story completions when matched to mood-responsive EEG group traits

Because our focus in the EEG study is on participants for whom the movie manipulation worked as intended, we conducted an additional “mood-matched” analysis of the story completion data. To achieve this, we removed the data of the 6 “best-mood” participants in the negative mood induction group (all scoring over 1.69 on the 10-item mood scale across 5 reading blocks), and the 6 “worst-mood” participants in the positive mood induction group (all scoring below 1.59), from our current analysis. Of the remaining 28 participants, those shown clips of Happy Feet reported being in a much better mood than those shown Sophie's Choice clips, in every block, as well as averaged across all five blocks [average mood score after HF: 2.12, after SC: 0.55; between-participants *F*_(1, 26)_ = 53.54, *MSE* = 0.32, *p* < 0.001]. Importantly, and as in the earlier all-participants analysis, participants in both groups produced equally biased story completions, with on average 90% (SD 4%, range 83–96%) bias-consistent story completions in the positive mood induction condition, and 91% (SD 4%, range 82–97%) in the negative mood induction condition [*F*_(1, 26)_ = 0.11, *MSE* = 17.09, *p* = 0.747].

Interestingly, participants in the positive mood induction group produced marginally longer sentence completions than participants in the negative mood induction group [6.3 vs. 5.6 words, sd = 1.15 vs. 0.81, range = 4.8–9.0 words vs. 4.1–6.6 words, respectively; *F*_(1, 26)_ = 3.12, *MSE* = 0.99, *p* = 0.089], for the exact same materials, and under the same task demands (“be short”). Also, across all 28 participants, self-reported mood across the five experimental blocks correlated reliably with mean completion length (Pearson *r* = 0.41, *p* = 0.032 two-tailed), whereas self-reported mood at the start of the experiment did not (Pearson *r* = −0.03, *p* = 0.873 two-tailed). This suggests that an experimentally induced bad mood causes people to be slightly less “wordy,” at least under current conditions.

## Discussion

In two language comprehension experiments, we used implicit causality phrases such as “Linda praised David because … ” to examine whether a lab-induced bad mood down-regulates the degree to which readers heuristically anticipate who will be talked about next. In the EEG study, results were as predicted, at least for readers whose self-reported mood changed in the intended way. When the moderately good mood with which these participants arrived at the lab was preserved, pronouns that were inconsistent with verb-based implicit causality elicited a clear processing cost, with the same ERP signature as reported by Van Berkum et al. (2007). When the same participants' mood deteriorated as a result of watching rather depressing Sophie's Choice clips, this signature of referential anticipation disappeared.

A decline in mood did not reliably affect ERP indices of early stimulus processing (N1 and P2), nor did it lead to a reliably smaller P600 response to syntactic anomalies. The absence of referential anticipation in a bad mood can therefore not be attributed to our participants tuning out, being distracted to such an extent that they no longer focused on the texts. Also, mood did *not* affect the use of the implicit causality heuristic when participants were asked to provide a continuation and were given enough time to think about it in a behavioral experiment. This suggests that the mood-induced failure to use this heuristic to anticipate reference in the EEG study is specific to the real-time demands of reading along with rapidly unfolding text, without additional task demands.

### What exactly did we manipulate?

Following earlier mood research available when we designed the study, we started out with a coarse valence model of mood as good or bad, and assessed the success of our manipulation on an aggregate mood scale involving 10 strongly valenced mood adjectives. However, the landscape of mood may well be more articulate and interesting than that (see Hamann, 2012; Shiota and Kalat, 2012, for discussion). For example, recent work suggests that although high-motivation negative affects like disgust, fear or anger tend to cause a narrower, more focused perspective, *low*-motivation negative affects such as sadness actually causes a broader spotlight of attention (Gable and Harmon-Jones, 2009, 2010).

The 15 additional state adjectives that we used as fillers in our self-report questionnaire can provide some information on what the movie clips did to our readers. EEG participants whose global mood, indexed by our 10-item valence scale, went down at least half a scale point upon viewing clips from Sophie's Choice instead of Happy Feet also reported feeling reliably more nervous, more afraid and less relaxed, as well as less motivated, less focused, less self-assured, and marginally less active. In the film ratings, Sophie's Choice was rated as reliably more exciting and interesting. But with respect to arousal aspects of the self-report, the relative impact of Sophie's Choice is somewhat ambiguous, with clear hints at increased arousal (increase in nervousness, fear and insecurity), but also some support for less arousal (decrease in motivation, and in a feeling of active engagement). Perhaps the hints at increased arousal are indicative of high motivational intensity with respect to events depicted in Sophie's Choice, whereas hints at decreased arousal reflect reduced motivational intensity for the reading task; this makes it difficult to relate our ratings to a single arousal or motivation dimension. The one clear message that these additional measures do convey is that, regardless of what the films did to their arousal, people felt less good after Sophie's Choice clips.

While our negative mood induction successfully caused the mood of these participants to go down in both experiments, positive mood induction did not make them feel any better than how they felt when they entered the lab. It seems that although such clips may come as a pleasant surprise in a psycholinguistic experiment, they don't *really* cheer people up. This is consistent with the fact that the ERP effect elicited by bias-inconsistent pronouns after positive mood induction is of similar size as the effect we obtained to the same materials without mood induction (Van Berkum et al., 2007).

We selected Sophie's Choice and Happy Feet to manipulate mood because these movies have frequently been used in mood research, and are known to induce sufficiently different moods. However, besides being cheerful or depressing, the two movies differed in several other ways. The most obvious difference is that whereas Happy Feet is an animation featuring talking penguins, Sophie's Choice is a realistic piece of human drama. Related, the events portrayed in the Happy Feet clips that we used are less complex than those in the Sophie's Choice clips, the traces of which can be seen in different complexity ratings given by our participants. We cannot exclude that such differences have an impact on the reading task, such as via fatigue, or depletion (Baumeister and Vohs, 2007). However, such an impact may actually be part and parcel of why Sophie's Choice puts you down, and Happy Feet does not. And part of the higher complexity rating of Sophie's Choice may well reflect the emotional complexity of depressing interpersonal interactions. For the time being, the most parsimonious interpretation of how participants were affected by our movie clips, supported by the overall 10-item mood scale as well as the common denominator in other self-rating differences, is that it changed their mood.

We focused on female readers whose self-reported mood changed in the intended way, as verified by a self-report global mood scale, and observed the predicted pattern of ERP results in this group. In our study, these readers also scored higher on the IRI Fantasy scale, which taps into their capacity or inclination to feel for, or feel along with, characters in fictional narrative (Davis, 1983). This suggests that the individual differences in our ERP results may simply reflect different degrees of sensitivity to the specific manipulation, a movie, and that the participants whose mood did not change in the intended way in response to Sophie's Choice might turn out to be more responsive to some other mood manipulation. We also suspect that an effective manipulation for *men*, whether Sophie's Choice, bad treatment by experimenters, or seeing one's favorite soccer team lose, will reveal a similar pattern of results. These predictions remain to be tested.

### Why does mood affect referential anticipation in reading?

What might be the underlying mechanism linking a bad mood to a failure to use the implicit causality heuristic in real-time language processing? Emotion is generally considered to involve the synchronous, interrelated recruitment of *several* brain-body systems (Damasio, 1996, 2010; Scherer, 2005; Frijda, 2007; Shiota and Kalat, 2012), including perceptual and cognitive systems needed for unconscious and conscious (re)appraisal of the situation, the autonomous nervous system, the neuro-endocrine system, the somatic nervous system involved in motor control, and various processes that allow people to become subjectively aware of at least some of their bodily changes, giving rise to “feelings.” Although slower-acting, less event-tied, and usually less intense (Scherer, 2005), changes in mood presumably involve a similarly rich set of changes, as such offering multiple sites via which cognition and action can be affected. In spite of the attractiveness of simple models, therefore, we think it is unlikely that mood simply involves adjustment of a single parameter of mind. Rather, the default hypothesis should be that mood involves the simultaneous tuning of *multiple* different parameters of our mental architecture, which collectively reconfigure perception, cognition, and action into particular modes. The language comprehension processes studied here also involve a complex orchestration of multiple systems, depending on such diverse operations as memory retrieval, sentence-level unification, dynamic situation modeling, anticipation, and context-dependent inferencing. The implication of all this complexity is that there is bound to be more than one route via which a change in mood can in principle bring about the processing effects we see in our EEG study. We discuss two plausible candidate scenarios, as well as their possible relation.

The first candidate involves mood-induced changes in the scope of associative memory retrieval. Relative to a good mood, a bad mood not only leads to narrower visuo-spatial attention, but also to a narrower “spotlight” of associative retrieval from long-term memory, interfering with access to more remote associations (Isen et al., 1985; Federmeier et al., 2001; Bolte et al., 2003; Rowe et al., 2007; Friedman and Förster, 2010). This effect may be mediated by increased inhibitory control exerted by medial prefrontal cortex over associative memory retrieval in medial temporal lobe structures (Bar, 2009). To account for the absence of referential anticipation in negative mood sessions of the EEG experiment, we need to assume that the implicit causality information associated with verbs like “praise” or “apologize” is sufficiently remotely associated to those verb forms to—at least under some conditions—fall outside the spotlight of retrieval. Furthermore, we have to assume that, given the same retrieval scope, the morpho-syntactic aspects of particular word forms needed for agreement checking are fully accessible. Although generated *ad-hoc*, these are not unreasonable assumptions: in the highly constrained domain of syntax, the plural feature associated with a word form such as “boys” could hardly be called a remote association, but probabilistic information that in “X praised Y because … ” constructions speakers are more likely to continue with information on Y than on X can be construed as “remote.” This makes it conceivable that in a sufficiently bad mood, implicit causality information falls outside of the spotlight of retrieval, whereas the agreement-relevant features needed for syntactic parsing are still sufficiently rapidly retrieved.

The second candidate scenario involves mood-induced changes in the effort invested in exploratory cognition. Mood has a direct impact on the willingness to invest in exploratory, or otherwise costly behavior. For example, a bad mood causes people to overestimate the steepness of a hill (Zadra and Clore, 2011), and the chronically negative mood we call depression is strongly associated with loss of energy and initiative (Davidson et al., 2002b). Such observations have led people to propose that mood directly signals the amount of resources available for exploratory behavior (Zadra and Clore, 2011), with negative mood signaling low energy, best spent on more conservative behaviors.

To make this bio-energetic explanation relevant to our results, one would need to assume that the anticipation of who will be referred to incurs a greater cost than syntactic parsing, and/or is somehow more optional. We are unaware of any study that directly confirms this, but note that syntactic parsing is typically conceived of as relatively resource-free (“a reflex,” Fodor, 1983), in part because it involves computations in a limited and highly structured domain. It is not inconceivable that keeping track of referents, and anticipating upcoming referents in the service of that, is somewhat more costly, possibly because it requires readers to reach out from the structured linguistic code, into a much more fuzzy, complex discourse world. Perhaps readers only do this when sufficiently motivated to do so, based on a trade-off between expected costs and benefits (cf. Sperber and Wilson, 1995). Such an account would be in line with the phenomenon—presumably familiar to all of us—that disinterest or fatigue induces shallower modes of reading, where syntax and semantics still seem to be doing their job, but do not seem to lead to a tangible, salient model of the situation described.

In essence, the bio-energetics account proposes that after having seen a negative-mood inducing film like Sophie's Choice, people are simply less inclined to invest in exploratory processing (possibly also because of “ego depletion,” Baumeister and Vohs, 2007), and consequently tune down heuristic referential anticipation. This account is consistent with recent behavioral evidence that a bad mood can attenuate the degree to which readers draw predictive inferences in text (Mirous and Beeman, 2012), and with N400 evidence that such a mood can also attenuate conceptual expectation in sentences and text (Federmeier et al., 2001; Chwilla et al., 2011, between-category violations; Pinheiro et al., 2013). However, for reasons currently not well understood, not all forms of anticipation are tuned by mood (e.g., Federmeier et al., 2001, within-category violations; Lai et al., 2012). In a way, this is reassuring, for with prediction increasingly viewed as a fundamental operating principle that underlies all of the brain's functions, including basic perception, motor control, emotion, and learning (Bar, 2011), one would not want a bad mood to shut down the whole system. Our working assumption, therefore, is that a bad mood can lead the system to cut back on only some aspects of anticipatory processing, perhaps because the associations involved are too remote, or because the process is otherwise more costly than affordable.

As for the latter, note that in the on-line situation of the EEG study, the time for prediction in sentences like “X praised Y because … ” is really short. Readers can only begin to generate implicit causality predictions at the verb, a more precise referential anticipation can only unfold upon reading about Y, and it needs to be in place right after “because.” With word durations ranging between 241 and 403 ms, and 106 ms separating each word, there is less than a second for the whole process to unfold. Furthermore, to the extent that a really precise implicit causality prediction actually requires the semantics of “because” (but see Majid et al., 2007; Pyykkönen and Järvikivi, 2010, for evidence that this is not always necessary), only a few hundred milliseconds is available. It is easy to imagine that such time-critical processing can suffer from slightly less investment of effort.

In a bio-energetic model, mood-induced adjustments in non-obligatory (e.g., exploratory) processing can be expected to depend on the precise trade-off between the costs and benefits of pursuing a certain course of physical or mental action. In a psycholinguistic experiment, such trade-offs will also be influenced by how the experiment is perceived, and what task demands are in place. The latter can explain why negative mood did not affect performance in the story completion experiment. After all, participants were specifically asked to come up with sensible continuations, a task that rendered the use of verb-based implicit causality information relevant and profitable.

We think it highly plausible that perceived relevance to the task at hand can counteract the bio-energetic impact of a bad mood. In line with this, note that in a bad mood, participants did come up with story completions that were about 10% shorter. This selective effect of mood makes sense, as (in contrast to the use of implicit causality information) wordiness itself is not relevant to the task at hand—depressed participants may well cut back on it. In general, we suspect there is a fruitful connection to be made between bio-energetic effects of mood on language processing on the one hand, and cost/benefit-oriented models of language comprehension such as Relevance Theory (Sperber and Wilson, 1995) on the other.

Finally, the bio-energetic account and the retrieval scope account can easily be combined into a single coherent story, where a bad mood reduces the inclination to cognitively explore, such that less effort is invested in a broad search in long-term memory, blocking the sufficiently rapid retrieval of information needed to extrapolate from, say, “David praised Linda because … ” to an expectation that Linda will soon be referred to. This is a parsimonious account for our findings. However, it does *not* rule out other causal scenarios. Mood is a complex phenomenon, and so is language comprehension.

### Mood and syntax

Our findings on syntactic anomaly suggest that lab-induced mood has little effect on people's sensitivity to subject-verb agreement violations: the size of the P600 effect did not reliably differ in its canonical latency range. However, the ERP traces do suggest that in a good mood, such agreement violations lead to somewhat earlier processing costs. Whether this should be interpreted as an earlier onset of the P600 effect or as something else is not entirely clear, because the scalp distributions of the early and late positivity are not entirely identical. Either way, this aspect of the data could be taken to suggest that, although obviously much more robust than referential anticipation, agreement checking is not *entirely* cost-free, and hence somewhat sensitive—at least in its timing—to people's willingness to invest effort.

The relative robustness of the late P600 effect in our study does conflict with results of Vissers et al. (2010), who found that negative mood induction caused a complete collapse of the P600 effect to subject-verb agreement violations. We see at least three differences between the two experiments that might account for these conflicting findings. First, whereas syntactic violations were part of slightly more engaging two- to four-sentence discourses in our study, they featured in isolated sentences in the Vissers et al. study. Second, we had asked our EEG participants to simply read for comprehension, while Vissers et al. told their participants that “questions would be asked about the sentences after the experiment.” Finally, our syntactic anomalies were embedded in syntactically and semantically very simple sentence fragments (e.g., “The boys_pl_ turns_sg^*^_ even the slightest difference of opinion into a bet”), whereas critical sentences in the Vissers et al. study always involved center-embedded subject- or object-relative clauses (e.g., The daughter_sg_ that about her parents_pl_ spoke_pl^*^_ burst_sg_ suddenly into tears; approximate translation), with distant agreement controllers as well as local agreement distractors. One possibility is that under these more complex syntactic conditions, and in view of the task demands imposed, syntactic processing becomes somewhat more effortful, and as such more sensitive to the bio-energetic consequences of a negative mood. Future research will have to resolve this issue.

## Conclusion

Although the underlying mechanisms are as yet not entirely understood, our studies reveal that mood can selectively tune certain parameters of the language processing architecture, as it operates in real time. In particular, the experiments show that under the conditions studied here, a lab-induced bad mood can down-regulate the extent to which readers rely on heuristics to rapidly anticipate who will be talked about next, while at the same time leaving syntactic parsing relatively unaffected. Does this generalize to the many other communicative arenas where joint attention plays a role? For instance, if we are in a negative mood, are we less inclined to resolve the many different names that populate classic Russian novels, or to anticipate what a museum tour guide is about to draw our attention to, with words or actual pointing? Possibly. What we do know for certain, however, is that we are all in a particular mood *all the time*, and that this affects our perception, cognition, and action in unobtrusive yet pervasive ways. Our findings show that, at least for those who were in the grip of a depressing movie, discourse-level language comprehension is no exception to the rule.

### Conflict of interest statement

The authors declare that the research was conducted in the absence of any commercial or financial relationships that could be construed as a potential conflict of interest.

## Footnotes

^1^Our logic critically hinges on the presence of a bias inconsistency ERP effect in particular latency ranges (expected around 400–700 ms, Van Berkum et al., 2007) in a positive mood. We examined the mood-induced attenuation of that specific ERP effect by testing for the mood by consistency interaction in the relevant latency ranges, rather than by solely relying on a non-significant bias inconsistency effect with negative mood (cf. Nieuwenhuis et al., 2011). However, in the absence of a reliable bias inconsistency ERP effect with positive mood in other latency ranges, a test for mood-induced attenuation in those other latency ranges would be ill-defined.

## Appendix: example stories

Shown are three examples that combine an implicit causality manipulation with a syntactic manipulation, as well as three examples that realize a syntactic manipulation only. Translations are approximate. Bias-consistent pronoun in **bold**, bias-inconsistent pronoun in **bold underlined**. Syntactically agreeing critical verbs are in *italics*, syntactically anomalous critical verbs are in *italics underlined*. The full Dutch item set is available from the first author.

(1a) Bias-consistent pronoun + syntactic agreement/anomaly
  Casper en Sophie waren al een aantal jaren goeie vrienden. Ze hadden elkaar leren kennen tijdens hun stage. Sophie waardeerde Casper omdat **hij** in die periode haar vaak had geholpen zonder dat hij daar zelf belang bij had. De vrienden *werkten_plural_/werkte*_*singular*_ nog steeds af en toe samen aan projecten.
  [Casper and Sophie had been close friends for several years. They got to know each other during an internship. Sophie appreciated Casper because **he** had often helped her in that time, even though it wasn't in his own interest. Every now and then, the friends still *collaborated_plural_/collaborated*_*singular*_ on projects].
(1b) Bias-inconsistent pronoun
  Casper en Sophie waren al een aantal jaren goeie vrienden. Ze hadden elkaar leren kennen tijdens hun stage. Casper waardeerde Sophie omdat **hij** in die periode veel hulp van haar had gekregen zonder dat zij daar zelf belang bij had.
  [Casper and Sophie had been close friends for several years. They got to know each other during an internship. Casper appreciated Sophie because **he** had received a lot of help from her in that time, even though it wasn't in her own interest].
(2a) Bias-consistent pronoun + syntactic agreement/anomaly
  Carice van Houten en Tom Cruise werkten aan een nieuwe film. Ze hadden samen het script bestudeerd. Tom irriteerde Carice omdat **hij** tijdens de opnames herhaaldelijk zijn tekst vergat. De andere actrices *dweepten_plural_/dweepte*_*singular*_ allemaal met hem, maar dat gevoel was zij inmiddels wel kwijt.
  [Carice van Houten and Tome Cruise were working on a new film. They had studied the script together. Tom annoyed Carice because **he** repeatedly forgot his lines during takes. The other actresses all *raved_plural_/raved*_*singular*_ about him, but as for her, she didn't feel like that at all anymore].
(2b) Bias-inconsistent pronoun
  Carice van Houten en Tom Cruise werkten aan een nieuwe film. Ze hadden samen het script bestudeerd. Carice irriteerde Tom omdat **hij** tijdens de opnames herhaaldelijk haar fouten moest corrigeren.
  [Carice van Houten and Tom Cruise were working on a new film. They had studied the script together. Carice annoyed Tom because **he** repeatedly had to correct her mistakes during takes].
(3a) Bias-consistent pronoun + syntactic agreement/anomaly
  Laurie en Jochem waren allebei dol op zoetigheid. Toen ze op paasochtend beneden kwamen, bleek de paashaas van chocola een oor te missen. Jochem bekende aan Laurie omdat **hij** wist dat ontkennen geen zin had. Hij *beloofde_singular_/beloofden*_*plural*_ ter compensatie een nieuwe paashaas te kopen zodra de winkels open waren.
  [Laura and John both really loved chocolate. When they got downstairs on Easter morning, the chocolate Easter Bunny turned out to miss an ear. John confessed to Laura because **he** knew there was no point in denying. To make up, he *promised_singular_/promised*_*plural*_ to buy a new Easter Bunny as soon as shops would be open].
(3b) Bias-inconsistent pronoun
  Laurie en Jochem waren allebei dol op zoetigheid. Toen ze op paasochtend beneden kwamen, bleek de paashaas van chocola een oor te missen. Laurie bekende aan Jochem omdat **hij** wist dat alleen zij het kon zijn geweest.
  [Laura and John both really loved chocolate. When they got downstairs on Easter morning, the chocolate Easter Bunny turned out to miss an ear. Laura confessed to John because **he** knew that only she could have done it].
(4) Syntactic agreement/anomaly
  Annabel en Tom hebben samen een krantenwijk. Annabel *doet_singular_/doen*_*plural*_ de linkerkant van de weg en Tom de rechterkant. Omdat er aan de linkerkant meer huizen staan is Tom vaak eerder klaar.
  [Annabel and Tom share a newspaper round together. Annabel *does_singular_/do*_*plural*_ the left side of the road and Tom does the right side. Because there are more houses on the left, Tom is often done first].
(5) Syntactic agreement/anomaly
  Vroeger nam mijn vader me vaak mee naar allerlei musea. We *praatten_plural_/praatte*_*singular*_ dan vaak uren voor een enkel schilderij. Hij wist overal wel iets interessants over te vertellen.
  [My dad used to take me to all kinds of museums. We often *talked_plural_/talked*_*singular*_ for hours about particular paintings. He always had something interesting to say, about anything].
(6) Syntactic agreement/anomaly
  Koen en Wilma gingen een avondje uit. Wilma *draaide_singular_/draaiden*_*plural*_ al uren rondjes voor de spiegel, ook al bleef Koen haar ervan verzekeren dat ze niet dik leek in die jurk. Ze stond daar nu al zo lang dat hij verging van de honger.
  [Ken and Wilma went out for dinner that night. For hours, Wilma *lingered_singular_/lingered*_*plural*_ in front of the mirror, even though Ken assured her again and again that she didn't look fat in that dress. She had been standing there for so long that, by now, he was starving].

## References

[B1] Baas M. De Dreu C. K. W. Nijstad B. A. (2008). A meta-analysis of 25 years of mood-creativity research: hedonic tone, activation, or regulatory focus? Psychol. Bull. 134, 779–806 18954157 10.1037/a0012815

[B2] Bar M. (2009). A cognitive neuroscience hypothesis of mood and depression. Trends Cogn. Sci. 13, 456–463 10.1016/j.tics.2009.08.009 19819753 PMC2767460

[B3] Bar M. (2011). Predictions in the Brain: Using Our Past to Generate a Future. Oxford: Oxford University Press

[B4] Baumeister R. F. Vohs K. D. (2007). Self-regulation, ego depletion, and motivation. Soc. Pers. Psychol. Compass 1, 1–13

[B5] Beukeboom C. J. Semin G. R. (2005). Mood and representations of behavior: the how and why. Cogn. Emot. 19, 1242–1251

[B6] Bless H. Bohner G. Schwarz N. Strack F. (1990). Mood and persuasion: a cognitive response analysis. Pers. Soc. Psychol. Bull. 16, 331–345 10.1177/0146167290162013

[B7] Bless H. Schwarz N. Clore G. L. Golisano V. Rabe C. Wölk M. (1996). Mood and the use of scripts: does a happy mood really lead to mindlessness? J. Pers. Soc. Psychol. 71, 665–679 8888596 10.1037//0022-3514.71.4.665

[B8] Bolte A. Goschke T. Kuhl J. (2003). Emotion and intuition: effects of positive and negative mood on implicit judgements of semantic coherence. Psychol. Sci. 14, 416–421 10.1111/1467-9280.01456 12930470

[B9] Chung G. Tucker D. M. West P. Potts G. F. Liotti M. Luu P. (1996). Emotional expectancy: brain electrical activity associated with an emotional bias in interpreting life events. Psychophysiology 33, 218–233 10.1111/j.1469-8986.1996.tb00419.x 8936391

[B10] Chwilla D. J. Virgillito D. Vissers C. T. (2011). The relationship of language and emotion: N400 support for an embodied view of language comprehension. J. Cogn. Neurosci. 23, 2400–2414 20849229 10.1162/jocn.2010.21578

[B11] Clark H. H. (1996). Using Language. Cambridge: Cambridge University Press 10.1017/CBO9780511620539

[B12] Clore G. L. Huntsinger J. R. (2007). How emotions inform judgment and regulate thought. Trends Cogn. Sci. 11, 393–399 10.1016/j.tics.2007.08.005 17698405 PMC2483304

[B13] Converse B. A. Lin S. Keysar B. Epley N. (2008). In the mood to get over yourself: mood affects theory-of-mind use. Emotion 8, 725–730 10.1037/a0013283 18837624

[B14] Damasio A. R. (1996). The somatic marker hypothesis and the possible functions of the prefrontal cortex. Philos. Trans. R. Soc. Lond. B Biol. Sci. 351, 1413–1420 10.1098/rstb.1996.0125 8941953

[B15] Damasio A. R. (2010). Self Comes to Mind: Constructing the Conscious Brain. New York, NY: Pantheon

[B16] Davidson R. J. Lewis D. A. Alloy L. B. Amaral D. G. Bush G. Cohen J. D. (2002a). Neural and behavioral substrates of mood and mood regulation. Biol. Psychiatry 52, 478–502 12361665 10.1016/s0006-3223(02)01458-0

[B17] Davidson R. J. Pizzagalli D. Nitschke J. B. Putnam K. (2002b). Depression: perspectives from affective neuroscience. Annu. Rev. Psychol. 53, 545–574 11752496 10.1146/annurev.psych.53.100901.135148

[B18] Davis M. A. (2009). Understanding the relationship between mood and creativity: a meta-analysis. Organ. Behav. Hum. Decis. Process. 108, 25–38 10.1016/j.obhdp.2008.04.00122409506

[B19] Davis M. H. (1983). Measuring individual differences in empathy: evidence for a multidimensional approach. J. Pers. Soc. Psychol. 44, 113–126 10.1037/0022-3514.44.1.113

[B20] de Vries M. (2008). Mood Matters in Judgment and Decision Making: Tuning in to Deliberation and Intuition. Dissertation, Radboud Universiteit Nijmegen.

[B21] de Vries M. Holland R. W. Chenier T. Starr M. J. Winkielman P. (2010). Happiness cools the warm glow of familiarity: psychophysiological evidence that mood modulates the familiarity-affect link. Psychol. Sci. 21, 321–328 10.1177/0956797609359878 20424063 PMC2948957

[B22] Egidi G. Gerrig R. J. (2009). How valence affects language processing: negativity bias and mood congruence in narrative comprehension. Mem. Cogn. 37, 547–555 10.3758/MC.37.5.547 19487747

[B23] Egidi G. Nusbaum H. C. (2012). Emotional language processing: how mood affects integration processes during discourse comprehension. Brain Lang. 122, 199–210 10.1016/j.bandl.2011.12.008 22325258

[B24] Featherstone C. R. Sturt P. (2010). Because there was a cause for concern: an investigation into a word-specific prediction account of the implicit-causality effect. Q. J. Exp. Psychol. 63, 3–15 10.1080/17470210903134344 19691006

[B25] Federmeier K. D. Kirson D. A. Moreno E. M. Kutas M. (2001). Effects of transient, mild mood states on semantic memory organization and use: an event-related potential investigation in humans. Neurosci. Lett. 305, 149–152 10.1016/S0304-3940(01)01843-2 11403927

[B26] Fiedler K. (2001). Affective influences on social information processing, in The Handbook of Affect and Social Cognition, ed Forgas J. P. (Mahwah, NJ: Erlbaum), 163–185

[B27] Fodor J. A. (1983). The Modularity of Mind. Cambridge, MA: MIT Press

[B28] Forgas J. P. (1995). Mood and judgment: the affect infusion model (AIM). Psychol. Bull. 117, 39–66 10.1037/0033-2909.117.1.39 7870863

[B29] Forgas J. P. (2007). Mood effects on memory, social judgments and social interaction, in Memory and mind: A festschrift for Gordon H. Bower, eds Gluck M. A. Anderson J. R. Kosslyn S. M. (Mahwah, NJ: Lawrence Erlbaum Associates), 262–281

[B30] Forgas J. P. Laham S. M. Vargas P. T. (2005). Mood effects on eyewitness memory: affective influences on susceptibility to misinformation. J. Exp. Soc. Psychol. 41, 574–588 10.1016/j.jesp.2004.11.005

[B31] Friedman R. S. Förster J. (2010). Implicit affective cues and attentional tuning: an integrative review. Psychol. Bull. 136, 875–893 10.1037/a0020495 20804240 PMC2933078

[B32] Frijda N. H. (2007). The Laws of Emotion. Mahwah, NJ: Erlbaum

[B33] Gable P. A. Harmon-Jones E. (2009). The blues broaden, but the nasty narrows: attentional consequences of negative affects low and high in motivational intensity. Psychol. Sci. 20, 1–5 20424047 10.1177/0956797609359622

[B34] Gable P. A. Harmon-Jones E. (2010). The motivational dimensional model of affect: implications for breadth of attention, memory, and cognitive categorization. Cogn. Emot. 24, 322–337 10.1080/02699930903378305

[B35] Garrett M. Harnish R. M. (2007). Experimental pragmatics: testing for implications. Pragmatics Cogn. 15, 65–90 10.1075/pc.15.1.07gar12485740

[B36] Garvey C. Caramazza A. (1974). Implicit causality in verbs. Linguist. Inq. 5, 459–464

[B37] Green M. C. (2004). Transportation into narrative worlds: the role of prior knowledge and perceived realism. Discourse Process. 38, 247–266 10.1207/s15326950dp3802_522994374

[B38] Gross J. J. Levenson R. W. (1995). Emotion elicitation using films. Cogn. Emot. 9, 87–108 10.1080/02699939508408966

[B39] Hagoort P. Brown C. M. Groothusen J. (1993). The syntactic positive shift (SPS) as an ERP measure of syntactic processing. Lang. Cogn. Process. 8, 439–483 10.1080/016909693084075859622187

[B40] Hamann S. (2012). Mapping discrete and dimensional emotions onto the brain: controversies and consensus. Trends Cogn. Neurosci. 16, 458–466 10.1016/j.tics.2012.07.006 22890089

[B41] Isen A. M. Johnson M. M. S. Mertz E. Robinson G. F. (1985). The influence of positive affect of the unusualness of word associations. J. Pers. Soc. Psychol. 48, 1413–1426 10.1037/0022-3514.48.6.1413 4020605

[B42] Kahneman D. (2011). Thinking, Fast and Slow. New York, NY: Farrar Straus and Giroux

[B43] Kiefer M. Schuch S. Schenck W. Fiedler K. (2006). Mood states modulate activity in semantic brain areas during emotional word encoding. Cereb. Cortex 17, 1516–1530 10.1093/cercor/bhl062 16926240

[B44] Koornneef A. W. Van Berkum J. J. A. (2006). On the use of verb-based implicit causality in setence comprehension: evidence from self-paced reading and eye tracking. J. Mem. Lang. 54, 445–465 10.1016/j.jml.2005.12.003

[B45] Lai V. T. Hagoort P. Van Berkum J. J. A. (2012). Mood and conflict in discourse, inPoster Presented at the 18th Annual Conference on Architectures and Mechanisms for Language Processing (Riva del Garda), 6–8

[B46] Levinson S. C. (2006). On the human “interaction engine”, in Roots of Human Sociality: Culture, Cognition and Interaction, eds Enfield N. J. Levinson S. C. (Oxford: Berg), 39–69

[B47] Majid A. Sanford A. J. Pickering M. J. (2007). The linguistic description of minimal social scenarios affects the extent of causal inference making. J. Exp. Soc. Psychol. 43, 918–932 10.1016/j.jesp.2006.10.016

[B48] Mirous H. J. Beeman M. (2012). Bilateral processing and affect in creative language comprehension, in The Handbook of the Neuropsychology of Language ed Faust M. (Oxford: Blackwell Publishing), 319–341

[B49] Nieuwenhuis S. Forstmann B. U. Wagenmakers E.-J. (2011). Erroneous analyses of interactions in neuroscience: a problem of significance. Nat. Neurosci. 14, 1105–1107 10.1038/nn.2886 21878926

[B50] Osterhout L. Kim A. Kuperberg G. R. (2012). The neurobiology of sentence comprehension, in The Cambridge Handbook of Psycholinguistics, eds Spivey M. Joannisse M. McCrae K. (Cambridge: Cambridge University Press), 365–389

[B51] Park J. Banaji M. R. (2000). Mood and heuristics: the influence of happy and sad states on sensitivity and bias in stereotyping. J. Pers. Soc. Psychol. 78, 1005–1023 10870905 10.1037//0022-3514.78.6.1005

[B52] Peeters F. P. M. L. Ponds R. H. W. M. Vermeeren M. T. G. (1996). Affectiviteit en zelfbeoordeling van depressie en angst. Tijdschr. Psychiatr. 38, 240–250

[B53] Pinheiro A. P. Del Re E. Nestor P. G. McCarley R. W. Goncalves O. F. Niznikiewicz M. (2013). Interactions between mood and the structure of semantic memory: event-related potentials evidence. Soc. Cogn. Affect. Neurosci. 8, 579–594 10.1093/scan/nss035 22434931 PMC3682442

[B54] Pratt N. L. Kelly S. D. (2008). Emotional states influence the neural processing of affective language. Soc. Neurosci. 3, 434–442 10.1080/17470910802188339 18633830

[B55] Pyykkönen P. Järvikivi J. (2010). Activation and persistence of implicit causality information in spoken language comprehension. Exp. Psychol. 57, 5–16 10.1027/1618-3169/a000002 20176549

[B56] Rottenberg J. Ray R. D. Gross J. J. (2007). Emotion elicitation using films, in The Handbook of Emotion Elicitation and Assessment, eds Coan J. A. Allen J. J. B. (London: Oxford University Press), 9–28

[B57] Rowe G. Hirsch J. B. Anderson A. K. (2007). Positive affect increased the breadth of attentional selection. PNAS 104, 383–388 10.1073/pnas.0605198104 17182749 PMC1765470

[B58] Scherer K. R. (2005). What are emotions? And how can they be measured? Soc. Sci. Inf. 44, 695–729

[B59] Schmitz T. W. De Rosa E. Anderson A. K. (2009). Opposing influences of affective state valence on visual cortical encoding. J. Neurosci. 29, 7199–7207 10.1523/JNEUROSCI.5387-08.2009 19494142 PMC6666470

[B60] Shiota M. N. Kalat J. W. (2012). Emotion. 2nd Edn Wadsworth: Cengage Learning

[B61] Sperber D. Wilson D. (1995). Relevance: Communication and Cognition. 2nd Edn Oxford: Blackwell

[B62] Subramaniam K. Kounios J. Parrish T. B. Jung-Beeman M. (2008). A brain mechanism for facilitation of insight by positive affect. J. Cogn. Neurosci. 21, 415–432 18578603 10.1162/jocn.2009.21057

[B63] Tomasello M. (2008). Origins of Human Communication. Cambridge, MA: MIT Press

[B64] Van Berkum J. J. A. Koornneef A. W. Otten M. Nieuwland M. S. (2007). Establishing reference in language comprehension: an electrophysiological perspective. Brain Res. 1146, 158–171 10.1016/j.brainres.2006.06.091 16916496

[B65] Van Steenbergen H. Band G. P. H. Hommel B. (2010). In the mood for adaptation: how affect regulates conflict-driven control. Psychol. Sci. 21, 1629–1634 20943936 10.1177/0956797610385951

[B66] Vissers C. T. Virgillito D. Fitzgerald D. A. Speckens A. E. Tendolkar I. van Oostrom I. (2010). The influence of mood on the processing of syntactic anomalies: evidence from P600. Neuropsychologia 48, 3521–3531 20696180 10.1016/j.neuropsychologia.2010.08.001

[B67] Watson D. Clark L. A. Tellegen A. (1988). Development and validation of brief measures of positive and negative affect: the PANAS scales. J. Pers. Soc. Psychol. 54, 1063–1070 3397865 10.1037//0022-3514.54.6.1063

[B68] Zadra J. R. Clore G. L. (2011). Emotion and perception: the role of affective information. Wiley Interdiscip. Rev. Cogn, Sci. 2, 676–685 10.1002/wcs.147 22039565 PMC3203022

